# Electrostatic Interactions in Asymmetric Organocatalysis

**DOI:** 10.1021/acs.accounts.3c00198

**Published:** 2023-07-06

**Authors:** Rajat Maji, Sharath Chandra Mallojjala, Steven E. Wheeler

**Affiliations:** Department of Chemistry, University of Georgia, Athens, Georgia 30602, United States

## Abstract

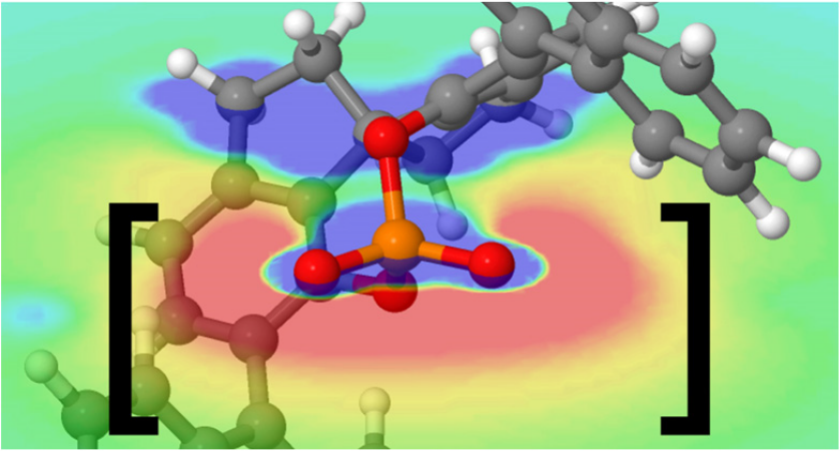

Electrostatic interactions are ubiquitous in catalytic systems and can be decisive in determining the reactivity and stereoselectivity. However, difficulties quantifying the role of electrostatic interactions in transition state (TS) structures have long stymied our ability to fully harness the power of these interactions. Fortunately, advances in affordable computing power, together with new quantum chemistry methods, have increasingly enabled a detailed atomic-level view. Empowered by this more nuanced perspective, synthetic practitioners are now adopting these techniques with growing enthusiasm.

In this Account, we narrate our recent results rooted in state-of-the-art quantum chemical computations, describing pivotal roles for electrostatic interactions in the organization of TS structures to direct the reactivity and selectivity in the realm of asymmetric organocatalysis. To provide readers with a fundamental foundation in electrostatics, we first introduce a few guiding principles, beginning with a brief discussion of how electrostatic interactions can be harnessed to tune the strength of noncovalent interactions. We then describe computational approaches to capture these effects followed by examples in which electrostatic effects impact structure and reactivity. We then cover some of our recent computational investigations in three specific branches of asymmetric organocatalysis, beginning with chiral phosphoric acid (CPA) catalysis. We disclose how CPA-catalyzed asymmetric ring openings of *meso*-epoxides are driven by stabilization of a transient partial positive charge in the S_N_2-like TS by the chiral electrostatic environment of the catalyst. We also report on substrate-dependent electrostatic effects from our study of CPA-catalyzed intramolecular oxetane desymmetrizations. For nonchelating oxetane substrates, electrostatic interactions with the catalyst confer stereoselectivity, whereas oxetanes with chelating groups adopt a different binding mode that leads to electrostatic effects that erode selectivity. In another example, computations revealed a pivotal role of CH···O and NH···O hydrogen bonding in the CPA-catalyzed asymmetric synthesis of 2,3-dihydroquinazolinones. These interactions control selectivity during the enantiodetermining intramolecular amine addition step, and their strength is modulated by electrostatic effects, allowing us to rationalize the effect of introducing *o*-substituents. Next, we describe our efforts to understand selectivity in a series of NHC-catalyzed kinetic resolutions, where we discovered that the electrostatic stabilization of key proton(s) is the common driver of selectivity. Finally, we discuss our breakthrough in understanding asymmetric silylium ion-catalyzed Diels–Alder cycloaddition of cinnamate esters to cyclopentadienes. The *endo*:*exo* of these transformations is guided by electrostatic interactions that selectively stabilize the *endo*-transition state.

We conclude with a brief overview of the outstanding challenges and potential roles of computational chemistry in enabling the exploitation of electrostatic interactions in asymmetric organocatalysis.

## Key References

- LuT.; WheelerS. E.Quantifying the Role of Anion−π Interactions in Anion−π Catalysis. Org. Lett.2014, 16, 3268–32712491552710.1021/ol501283u.^1^ This work examined the role of anion−π interaction in the first rerported example of anion−π catalysis, finding that these electrostatics-dominated noncovalent interactions actually increase the reaction barrier.
- SeguinT. J.; WheelerS. E.Electrostatic Basis for Enantioselective Brønsted-Acid-Catalyzed Asymmetric Ring Openings of meso-Epoxides. ACS Catal.2016, 6 (4), , 2681–2688.^2^ This paper provides a clear example where the stereoselectivity of a chiral phosphoric acid catalyzed reaction is not due to steric effects, but instead can be attributed to the preferential electrostatic stabilization of the transition state leading to the major stereoisomer.
- MajiR.; WheelerS. E.Importance of Electrostatic Effects in the Stereoselectivity of NHC-Catalyzed Kinetic Resolutions. J. Am. Chem. Soc.2017, 139, 12441–124492882316610.1021/jacs.7b01796.^3^ This publication shows the importance of electrostatic stabilization in NHC-catalyzed kinetic resolutions and the role of protic additives.
- SeguinT. J.; WheelerS. E.Stacking and Electrostatic Interactions Drive the Stereoselectivity of Silylium-Ion Asymmetric Counteranion-Directed Catalysis. Angew. Chem. Int. Ed.2016, 55, 15889–1589310.1002/anie.20160909527860103.^4^ This work highlights the importance of the heterogeneous electrostatic effect in controlling the diastereoselectivity in asymmetric silylium-based, counteranion-catalyzed Diels–Alder reactions.

## 1 Introduction

The past decade has seen considerable advancement in our understanding and appreciation of electrostatic interactions in organic systems. These interactions are pivotal in many stereoselective transformations, yet the difficulty of quantifying electrostatic interactions within transition state (TS) structures without detailed computational study prevents us from harnessing their full potential. Nevertheless, computational chemistry has facilitated a growing understanding of how subtle changes in electrostatic interactions can impact everything from conformation to reactivity and stereoselectivity, enabling the development of improved catalysts and even new reactions. In this Account, we offer a brief overview of noncovalent interactions whose strength can be tuned through electrostatic effects and chronicle our journey in this area over the past decade, including selected examples from other groups, while highlighting outstanding challenges. We particularly consider our contributions to elucidating physical aspects of electrostatic interactions, emphasizing the fundamental principles underlying electrostatic stabilization or destabilization, as illustrated through organocatalytic reactions, where they contribute critically.

We begin by discussing the role of electrostatic interactions in tuning the strength of noncovalent interactions and then outline the various computational approaches used to study them in organocatalysis. Building on these methods and principles, we describe selected examples where electrostatic interactions impact structure, reactivity, and selectivity in organocatalysis, followed by examples where knowledge of electrostatic interactions has facilitated the rational design of catalysts and reactions. We conclude by drawing attention to some outstanding challenges.

## 2 Brief Overview of Electrostatically Driven Interactions and Their Modulators

“Electrostatic interaction” refers to the Coulombic interaction of fixed charge distributions. This could include charge–charge, charge–dipole, dipole–dipole, etc. interactions. While the characterization of electrostatic interactions in molecular systems typically relies on quantum chemical methods (e.g., density functional theory) to derive electron distributions within molecules, the electrostatic interactions themselves are purely classical in nature and are therefore straightforward to understand.

**Figure 1:**
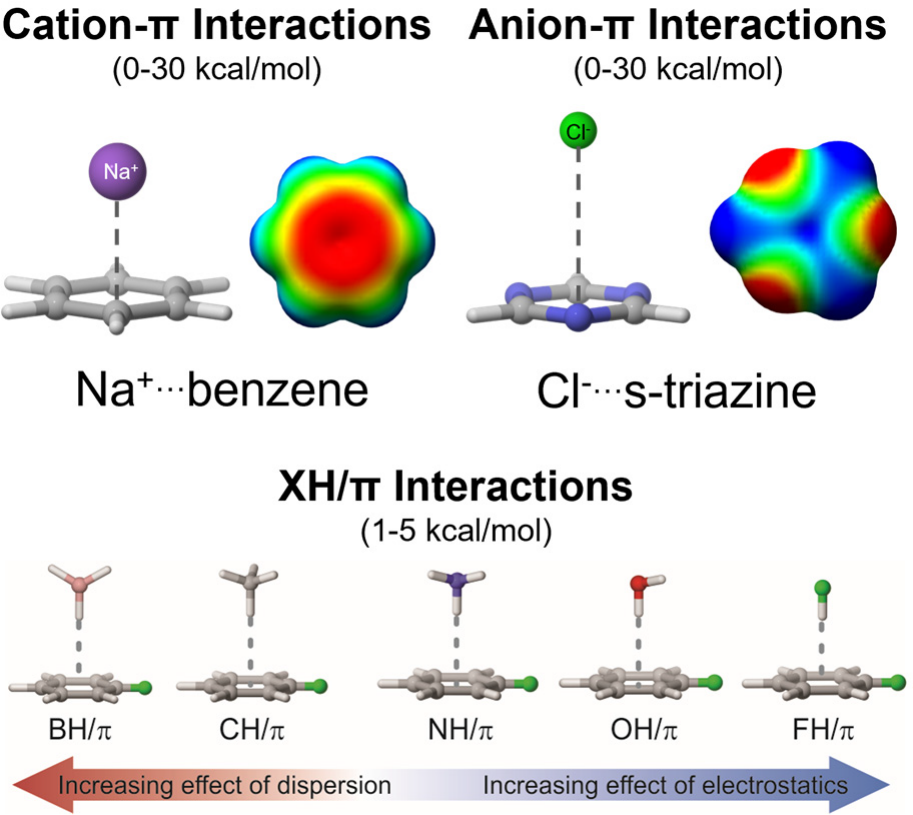
Examples of noncovalent interactions whose strengths can be tuned through electrostatic interactions, along with approximate ranges of interaction strength for small model systems.^5−7^

Electrostatic interactions can play a key role in many of the “named” noncovalent interactions with which chemists are familiar (Figure 1), sometimes providing the dominant attractive component. Perhaps the most obvious examples are cation−π and anion−π interactions, which are attractive interactions between atomic or polyatomic cations and anions and the face of an aromatic ring, respectively. While other effects contribute to these interactions, they are primarily electrostatic in origin. For other noncovalent interactions, the relative contribution of electrostatic effects can vary considerably. For instance, for XH/π interactions, which are interactions between any X–H bond and the face of an arene, electrostatic effects become increasingly important as the electronegativity of X increases. That is, OH/π and FH/π interactions are dominated by electrostatics, whereas CH/π interactions are driven mainly by dispersion effects.

Electrostatic interactions provide a simple means of tuning the interaction strength. In the case of noncovalent interactions involving aromatic rings, this involves modulation of the arene electrostatic potential (ESP) through the introduction of substituents^5,6^ and/or heteroatoms.^8−10^ For example, even though stacking interactions primarily result from dispersion interactions, stacking strength can be tuned over a large range by modulating the electrostatic potentials of one or both of the interacting arenes. As an example, the stacking interaction of 1,2,3,4-tetrazine with toluene is nearly twice as favorable as that of benzene with toluene. This is due almost entirely to a more favorable electrostatic contribution in the former case.^11^ Electrostatic effects are even more pronounced in stacking interactions, in which one arene bears a formal charge (i.e., π-π^+^ interactions). While it is common to ascribe changes in the ESP above the face of an arene to changes in the distribution or density of π-electrons, computations do not support this. Instead, we have shown^12^ that the dominant effect of substituents on the ESPs of arenes is through space, not through π-resonance. Similarly, the dramatic effect of heteroatoms on arene ESPs (e.g., compare the ESPs of benzene and 1,3,5-triazine in Figure 1) is not due to the π-electrons but instead arises from the rearrangement of charges within the molecular plane.^8^

Hydrogen bonds are an important class of noncovalent interactions whose strength can be modulated through electrostatic effects, and many stereoselective organocatalytic transformations hinge on the relative strength of one or more H-bonding interactions. These include conventional cases such as OH···O and NH···O hydrogen bonds but also nonclassical CH···O interactions.^13^ While these interactions are not purely electrostatic in origin, one can often explain the relative strength of two geometrically similar XH···Y interactions by considering the electrostatic potential experienced by the proton, which will bear a partial positive charge. These effects can be particularly pronounced in proton transfer reactions because such protons bear considerable partial positive charges.

## 3 Computational Tools to Probe Electrostatic Interactions in Catalysis

**Figure 2:**
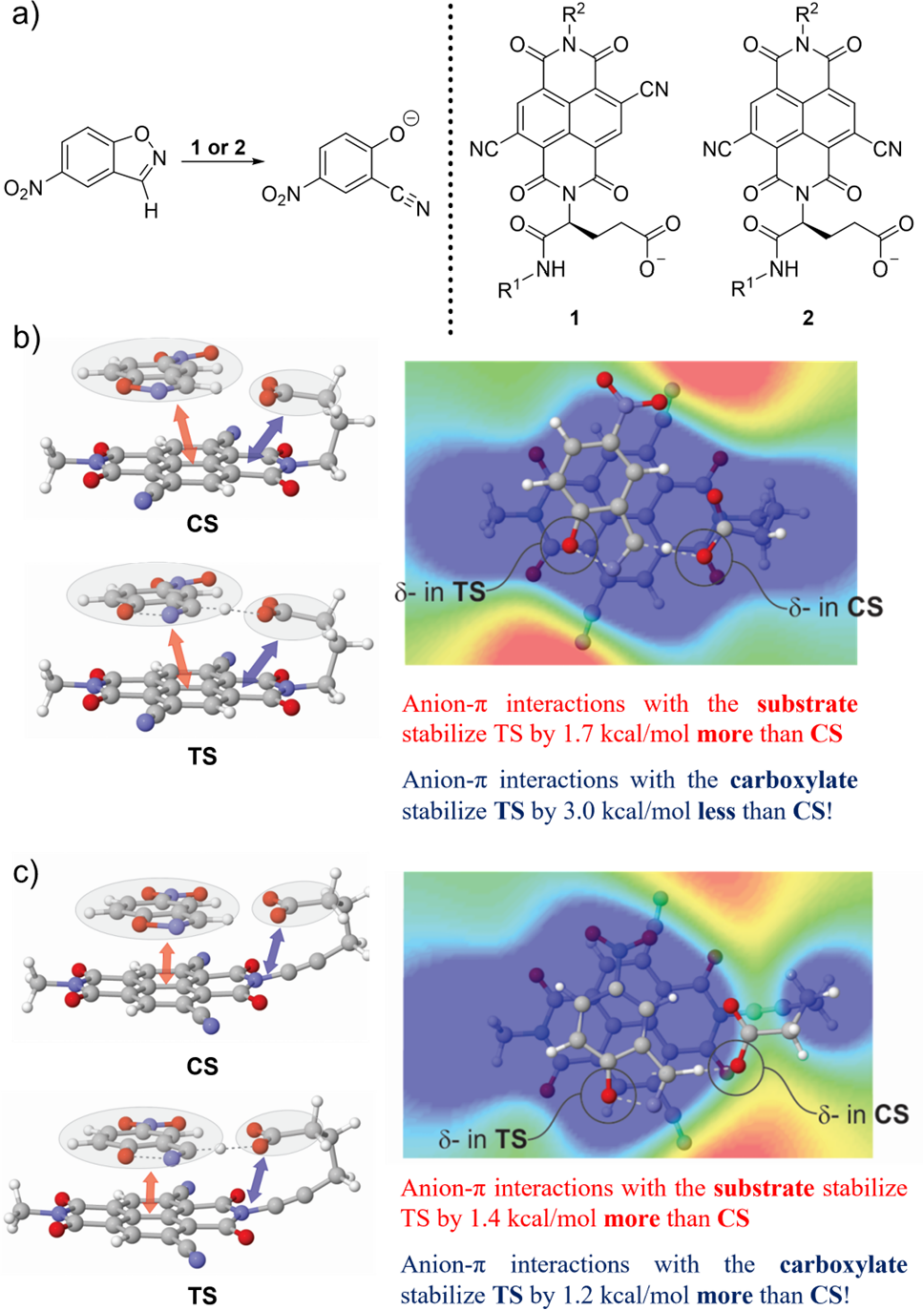
a) Kemp elimination studied by Matile and co-workers with the original catalyst from Matile et al.^28,29^ (**1**) and redesigned catalyst from Lu and Wheeler^1^ (**2**) b) and c) summary of the quantification of anion-π interactions in **CS** and **TS** for **1** and **2**, respectively, along with the ESPs due to the NDI component of the catalysts in the plane of the catalytic base and substrate. Portions adapted with permission from ref (1). Copyright (2014) American Chemical Society.

There are numerous computational approaches available to quantify electrostatic effects. Chief among these are computed ESPs (see examples in Figure 1). In these familiar plots, each point on a molecular surface (typically an electron density isosurface) is colored according to the ESP value at that position. Regions with negative ESP (typically colored red) will stabilize positive charges, whereas positive ESP regions (typically blue) will stabilize negative charges. Dougherty and co-workers^14^ established the utility of ESP plots for qualitative and quantitative prediction and analysis of cation−π interactions and graphical ESP representations have proved invaluable for modeling and understanding electrostatically driven noncovalent interactions. However, it is important to remember that the electrostatic interaction between two molecules is not given by the interaction of their respective ESPs! Instead, one must consider the distribution of charges (typically approximated as atomic partial charges) of one molecule interacting with the ESP due to the other molecule at the positions of these charges. Thus, we have found it far more fruitful to plot the ESP of one molecule in one or more planes containing key charged atoms of the other molecule (e.g.; see Figure 2b). One can approximate the total electrostatic interaction as the sum of the product of partial atomic charges of one molecule with the ESP of the other molecule evaluated at these positions. The results from such analyses will depend on the choice used to devise the atomic partial charges, but in general provide a reasonable estimate of electrostatic interactions with the further benefit of providing atomic-level detail.

More qualitatively, molecular dipole and quadrupole moments are often computed and used to rationalize inter- and intramolecular noncovalent interactions. While such analyses can be useful, *local* multipole moments are generally more important for understanding close-contact interactions than the molecular values. For example, while a symmetric molecule such as *para*-benzoquinone has no molecular dipole, the large local dipoles associated with each carbonyl group can engage in strong electrostatic interactions with other nearby molecules. This becomes increasingly important for larger molecules, because the leading term in the molecular multipole expansion becomes an increasingly poor predictor of the total electrostatic interaction as the size of the interacting molecules exceeds the distance between molecules.

Other, more specialized tools are available that can be used to quantify noncovalent interactions, including those with large electrostatic components. For example, atoms-in-molecules (AIM) provides a way to quantify polar interactions,^15^ while NCI analysis provides a qualitative means of identifying dispersion and steric interactions.^16^ Cheng, Houk, and co-workers^17^ used AIM to understand stereodifferentiation in squaramide-catalyzed asymmetric Michael addition of indoles to α-ketophosphonates, identifying an additional stabilizing CH···O interaction in the transition state (TS) leading to the major stereoisomer. Natural bond orbital (NBO) analysis is another widely used technique for quantifying interactions from an orbital perspective. For instance, Dudding and co-workers^18^ used NBO analyses to explain the preferred substrate binding mode and origin of stereoselectivity in an organocatalyzed aza-Henry reaction. Finally, symmetry adapted perturbation theory (SAPT)^19−22^ can provide robust predictions of interaction energies for nonbonded complexes and further decompose these energies into physically meaningful components, including electrostatic effects. We used SAPT to understand the fluxionality of chiral DMAP-catalyzed kinetic resolutions (*vide infra*).^23^ Atomic-SAPT (A-SAPT)^24^ and functional group-SAPT (F-SAPT)^25^ provide further opportunity to quantify electrostatic (and other) interactions at the group of individual atoms and functional groups, respectively, as demonstrated by Bakr and Sherrill^26^ in their analysis of electrostatic control in the enantioselective addition of allyl and allenyl organoboron reagents to fluorinated ketones.^27^

Practitioners must be aware that the methods mentioned above all have limitations and can be sensitive to the system under study. Thus, it is typically desirable to quantify interactions using several different methods to properly validate conclusions. For example, we relied on multiple techniques to understand the selectivity of *N*-heterocyclic carbene (NHC)-catalyzed kinetic resolution (KR) of cyclic diols,^3^ including a fragmentation approach and subsequent quantification using AIM, NBO, and direct estimation of electrostatic stabilization through computed ESPs and partial charges.

## 4 Electrostatic Effects on Structure and Activity

Armed with knowledge of noncovalent interactions for which electrostatic effects can play key roles, as well as techniques for their quantification, we turn to examples of organocatalytic reactions in which electrostatic effects impact either structure or catalytic activity.

First, our work on Matile’s anion-π-catalyzed Kemp elimination^28,29^ (Figure 2a) highlighted the importance of quantifying the role of electrostatic interactions in both the reactant and TS structure.^1^ In this model reaction, the deprotonation of a nitrobenzoxazole by a catalytic base triggers ring opening and formation of the cyanophenolic product. By engineering this reaction to occur over the face of a naphthalene diimine (NDI) through the use of a tethered carboxylate (**1**, Figure 2a), Matile *et al*. demonstrated^28,29^ the feasibility of using anion-π interactions to achieve rate acceleration. The rate of this reaction will depend on the free energy difference between the transition state (**TS**) and the catalyst substrate complex (**CS**). We showed that anion-π interactions are operative in both **CS** and **TS**. In the former, the anion is localized on the carboxylate, whereas in the latter, it is delocalized across the substrate. More importantly, quantification of these anion-π interactions revealed that they were more stabilizing in **CS** than in **TS** (see Figure 2b), meaning that the net result of anion-π interactions was an overall increase in the reaction barrier. In other words, we found that while **1** undoubtedly catalyzes reaction 1, anion-π interactions were not responsible for the observed rate acceleration. This can be further understood by considering the ESP due to the NDI in the plane of the carboxylate and substrate (Figure 2b). Going from **CS** to **TS**, the negative charge moves from one region of the positive ESP to another. While a positive ESP indicates stabilization of negative charge, because the ESP is uniformly positive across this region the electrostatic stabilization of the negative charge is roughly equal regardless of whether it is centered on the carboxylate or delocalized onto the substrate!

Although we did not find evidence of anion-π-induced barrier lowering for **1**, we devised a modified catalyst (**2**) for which we predict significant rate acceleration that can be attributed to anion-π interactions. By introducing an ethynyl linkage, the carboxylate is shifted to a region above the periphery of the NDI that has a more negative ESP. This effect is enhanced by moving one of the nitrile groups. The result is that there is now a strong electrostatic driving force for the migration of negative charge from the carboxylate to the substrate. This can be seen quantitatively in Figure 2c. For **2**, anion-π interactions of both the substrate and carboxylate are more favorable in **TS** and **CS**, resulting in a significant lowering of the energy barrier. Viewed another way, catalyst **2** enhances the basicity of the carboxylate by placing it in a less stabilizing electrostatic environment, thus providing a strong electrostatic driving force for the proton transfer.

**Figure 3:**
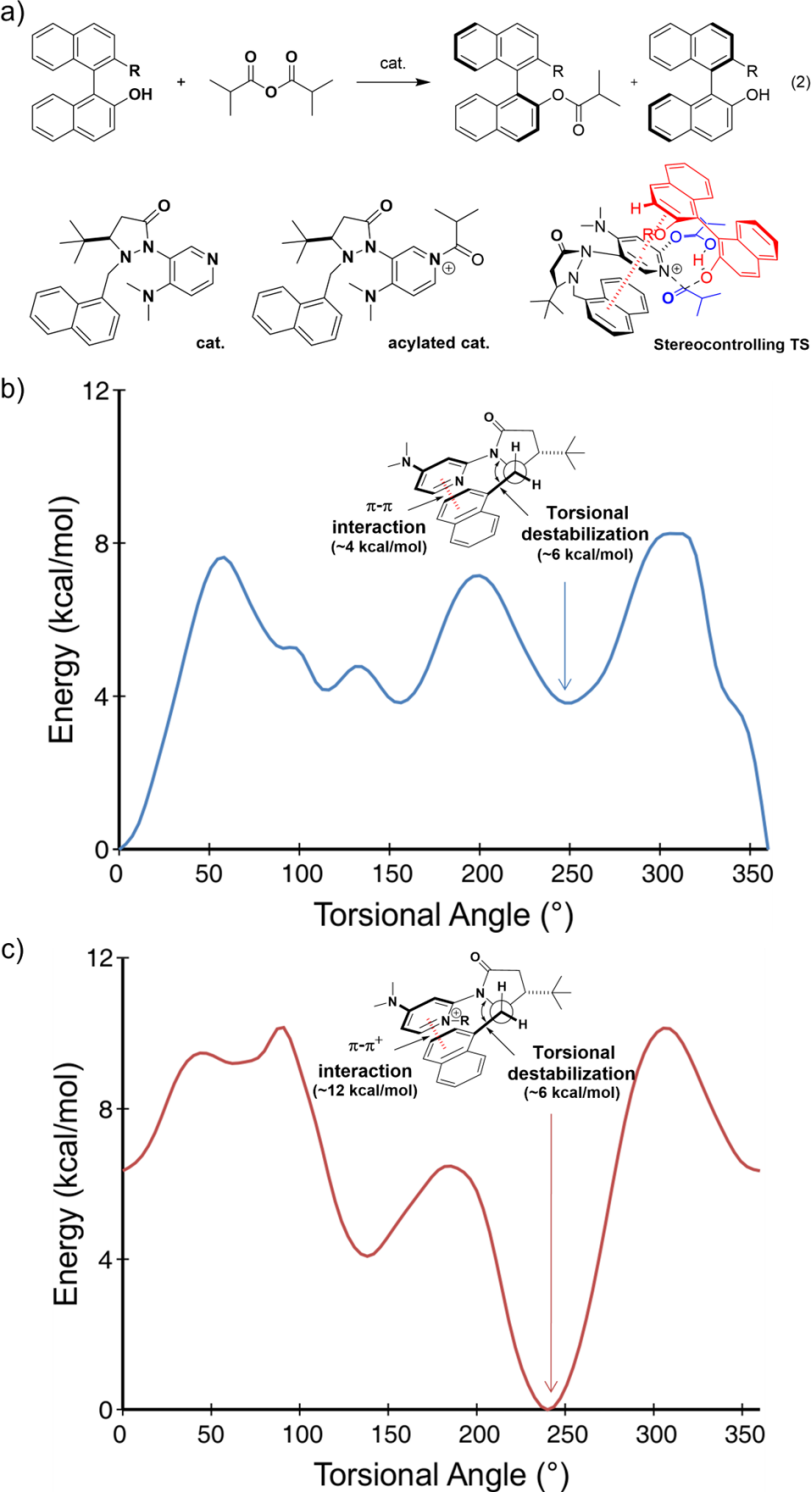
a) DMAP-catalyzed kinetic resolution of biaryls from Sibi et al.^30^ along with torsional analysis of the unacylated b) and acylated c) catalyst. π-π^+^ stacking interactions between the naphthyl group and pyridinium rigidify the catalyst and give rise to the conformaiton that proved vital for stereoselectivity.

As a second, more subtle example, we identified^23^ a central role of π–π^+^ interactions in the structural organization of DMAP catalysts for the kinetic resolution of axially chiral biaryls developed by Sibi et al.^30^ (reaction 2, Figure 3). Although Sibi and co-workers^30^ suggested that these catalysts are fluxional, we showed that the fluxionality in the ground state is lost during the course of the reaction. While multiple conformers are accessible for the inactive, unacylated catalyst, upon *N*-acylation, a previously unfavorable conformation becomes dominant (see Figure 3b and c). SAPT analysis indicated that this conformational bias in the acylated catalyst stems from electrostatic interactions between the naphthyl group and the pyridinium that overpower the intrinsic torsional destabilization of this conformer; prior to acylation, the corresponding neutral stacking interaction is too weak to overcome the torsional strain. Notably, the conformer exhibited by the acylated catalyst enables the formation of a sandwichlike π–π^+^–π stacking interaction in the stereocontrolling TS structures that proved vital for the facial selectivity of the acyl transfer.

## 5 Impact of Electrostatic Interactions on Stereoselectivity

Next, we consider three classes of organocatalysts for which we have found electrostatic interactions to play a key role in selectivity, either increasing or decreasing the free energy difference for the stereocontrollable TS structures.

### 5.1 The Heterogeneous Electrostatic Environment of Chiral Phosphoric Acids

We have extended considerable effort to understanding the origins of stereoinduction in chiral phosphoric acid (CPA)-catalyzed reactions.^2,31−36^ Over the past decade, CPAs have become widely used organocatalysts.^37^ While conventional stereoselectivity explanations invoke steric factors, including shape complementarity between the reactant(s) and the chiral binding pocket of the catalyst, computational studies have offered a more nuanced picture wherein stereocontrol often hinges on the interplay of attractive and repulsive noncovalent interactions. Sometimes, similar noncovalent interactions are present in competing stereocontrolling TS structures, and it is subtle differences in electrostatic interactions that tip the scales one way or the other. The importance of electrostatics in CPA catalysis has been echoed multiple times in computational studies, with additional experimental support from Gschwind and co-workers.^38^ Electrostatic stabilization by CPAs and related systems relies in many cases on their ability to engage substrates via classical (OH···X and NH···O) or nonclassical (CH···O) hydrogen bonds.^39−42^ The chiral binding pocket of CPAs offers a highly heterogeneous electrostatic environment, and we have found that the selectivity of some reactions can be understood based on the precise placement of key substrate protons within this environment in the stereocontrolling TS structures.

**Figure 4:**
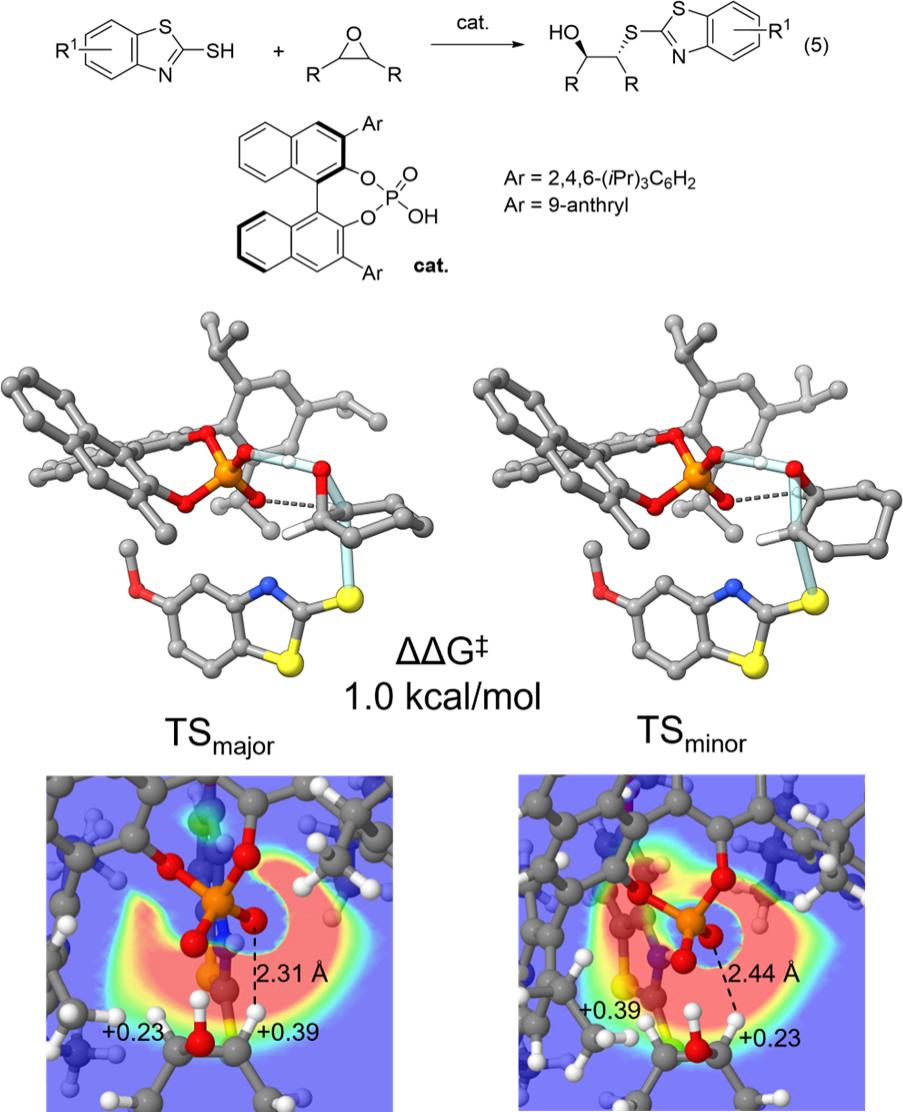
CPA catalyzed asymmetric ring-openings of cyclohexane oxide from Sun et al.^43^ Representative TS structures leading to the major and minor stereoisomers are shown along with the ESP of the catalyst in the plane of the two CH groups of the epoxide and NPA charges of these CH groups. Selected hydrogens are removed for clarity.

Our first discovery^2^ in this area involved CPA-catalyzed asymmetric epoxide ring openings from Sun et al.^43^ (reaction 5, Figure 4) and related reactions from List et al.^44,45^ We found that the narrow binding pocket of the catalyst resulted in the substrate adopting nearly identical orientations in the TS structures, leading to the major and minor stereoisomers (see Figure 4), resulting in nearly identical steric interactions. The difference in energy between these transition states is due to structurally similar CH···O hydrogen bond in both TS structures, as was previously reported for similar reactions by Ajitha and Huang.^46^ For the TS leading to the major stereoisomer, this interaction involves the CH undergoing nucleophilic attack, which is not the case for the minor TS. Due to the buildup of significant positive charge on this CH group during the reaction (see Figure 4), the major TS enjoys significantly more electrostatic stabilization through the interaction with the phosphoryl oxygen of the catalyst (Figure 4d). Viewed another way, the narrow binding pocket of the catalyst positions the two epoxide carbons in different electrostatic environments, leading to a strong preference for nucleophilic attack of one over the other.

**Figure 5:**
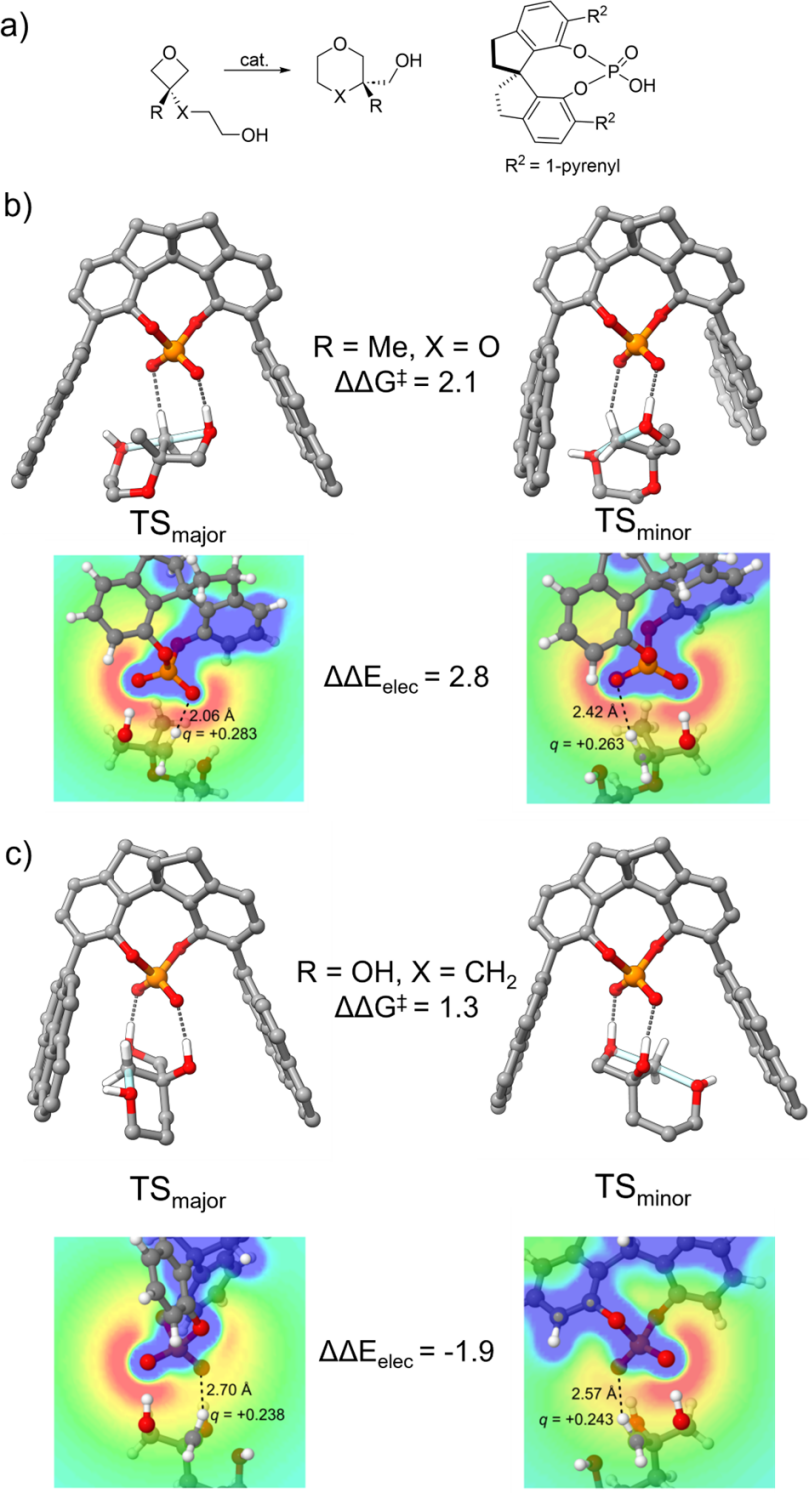
a) CPA-catalyzed intramolecular oxetane desymmetrization from Sun et al.;^47^ b) and c) quantifying the elelectrostatic controbution to the energy difference between the minor and major stereocontrolling TS structures (ΔΔE_elec_, in kcal/mol). Selected hyrogens were removed for clarity. Portions adapted with permission from ref (33). Copyright (2019) John Wiley and Sons.

**Figure 6:**
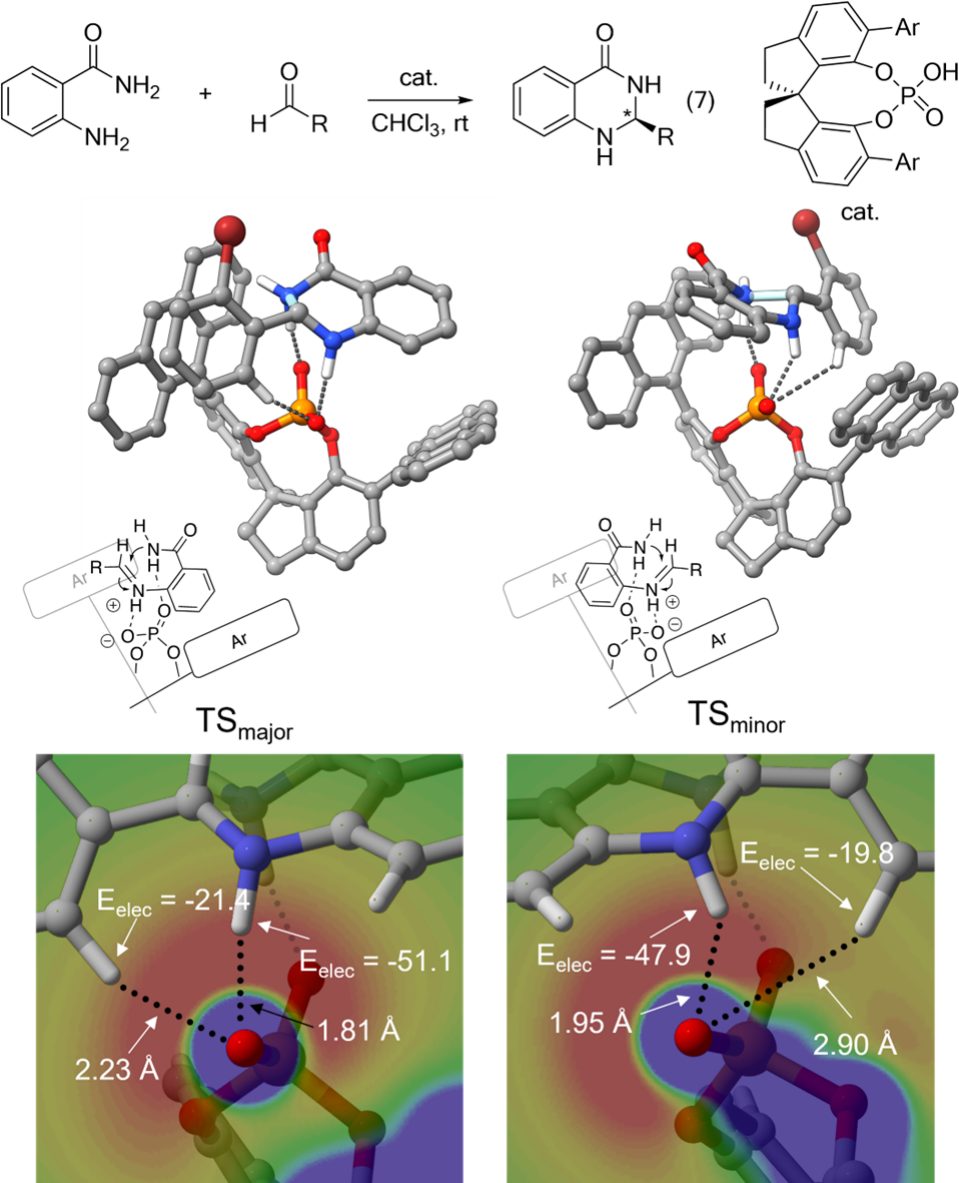
CPA-catalyzed synthesis of 2,3-dihydroquinazolinones from Huang and co-workers.^48^ Representative TS structures leading to the major and minor stereoisomers are shown (for R = *o*-BrPh), along with the ESP of the catalyst in the plane of the CH and NH bonds of the substrate for TS_major_ and TS_minor_, along with the atomic charges on the hydrogens and resulting electrostatic interaction (*E*_elec_, in kcal/mol). Selected hydrogens removed for clarity. Portions adapted with permission from ref (35). Copyright (2020) American Chemical Society.

In subsequent work, in collaboration with Champagne and Houk,^33^ we observed contrasting substrate-dependent electrostatic influences on intramolecular oxetane desymmetrizations from Sun et al.^47^ (reaction 6, Figure 5a). For oxetanes with nonchelating groups (R = Me, Figure 5b), the TS structures leading to both the major and minor stereoisomers feature a CH···O interaction involving the methylene group undergoing nucleophilic attack. The superior selectivity observed for such substrates was attributed to the more stabilizing interaction in the TS leading to the major stereoisomer. This can be understood in terms of the electrostatic interactions of this group with the phosphoryl oxygen of the catalyst. From Figure 6b, it can be seen that while the protons in the major and minor TS have similar positive charges, the former is in a more favorable electrostatic environment. The net result is that this single proton contributes 2.8 kcal/mol to preferential stabilization of TS_major_ over TS_minor_.

In contrast, introducing a chelating group (R = OH, Figure 4c) induces a different binding mode that is primarily stabilized by two OH···O hydrogen bonds. Consequently, the α-CH is distant from the phosphoric acid functionality in both the major and minor TS structures. We found that the electrostatic stabilization of this proton now favors the minor stereoisomer by 1.9 kcal/mol, contributing to the overall lowest selectivity of this substrate.

Finally, while exploring the origin of enantioselectivity in the asymmetric synthesis of 2,3-dihydroquinazolinones using SPINOL-derived CPAs from Huang and co-workers^48^ (reaction 7, Figure 6), we identified a key role for hydrogen bonds with the phosphate group of the catalyst whose strength varies depending on substrate positioning within the electrostatic environment of the catalyst.^35^ The enantiodetermining intramolecular amine addition step was analyzed computationally for reactions featuring 12 aryl R groups, giving excellent agreement with experiment (representative stereocontrolling TS structures are shown in Figure 6). Introducing *o*-substituents on the aryl group preferentially enhances the NH···O and CH···O interactions in the major TS over the minor TS, allowing the observed major isomer to be rationalized in terms of both electrostatics and geometry. The strength of these interactions is modulated by the ESP due to the catalyst at the positions of the two protons, establishing the feasibility of precisely controlling the selectivity in CPA-catalyzed reactions by tuning electrostatic interactions. This effect is maximized in the case of R = *o*-BrPh. In this case, the electrostatic stabilization of the NH and CH protons is 3.2 and 1.6 kcal/mol more favorable in TS_major_ than TS_minor_, respectively, contributing significantly to the observed 98% ee.

In general, the ESP within the binding pocket of a deprotonated CPA catalyst is dominated by the effect of the two oxygen atoms. The negative electrostatic potentials arising from these atoms decay rapidly with the distance, leading to a heterogeneous electrostatic environment. The result, as shown by the above examples, is that even small differences in the positions of protons can lead to large changes in the electrostatic stabilization. The steric demands of the flanking aryl groups of these catalysts have traditionally been the focus in CPA design, and the stereoselectivity of many CPA-catalyzed reactions can be explained solely in terms of the shape of the binding pocket and substrate. However, in the above examples, these sterically demanding groups play a slightly different role—controlling the precise orientation of the substrate within the chiral electrostatic environment of the catalyst. There is a potential to also use these aryl groups to further tune the ESP within the CPA binding pocket, and the introduction of substituted arenes or heteroarenes at these positions could be a potentially fruitful avenue for the development of CPA catalysts that more fully exploit electrostatic effects.

### 5.2 NHC Organocatalysis

NHCs are powerful organocatalysts capable of steering numerous challenging enantioselective transformations.^49^ An early electrostatically guided example was provided by the Rovis and Houk groups’ study of asymmetric intermolecular Stetter reactions,^50^ where stereoselectivity could be modulated by strategically tuning the electrostatic environment surrounding the NHC catalyst. In a study of NHC-catalyzed [4 + 2] cycloadditions, Kozlowski, Bode, and co-workers^51^ underscored the importance of an oxyanion steering mechanism that maximizes electrostatic interactions, while Scheidt, Cheong, and co-workers^52^ and Schoenebeck et al.^53^ have documented similar effects.

**Figure 7:**
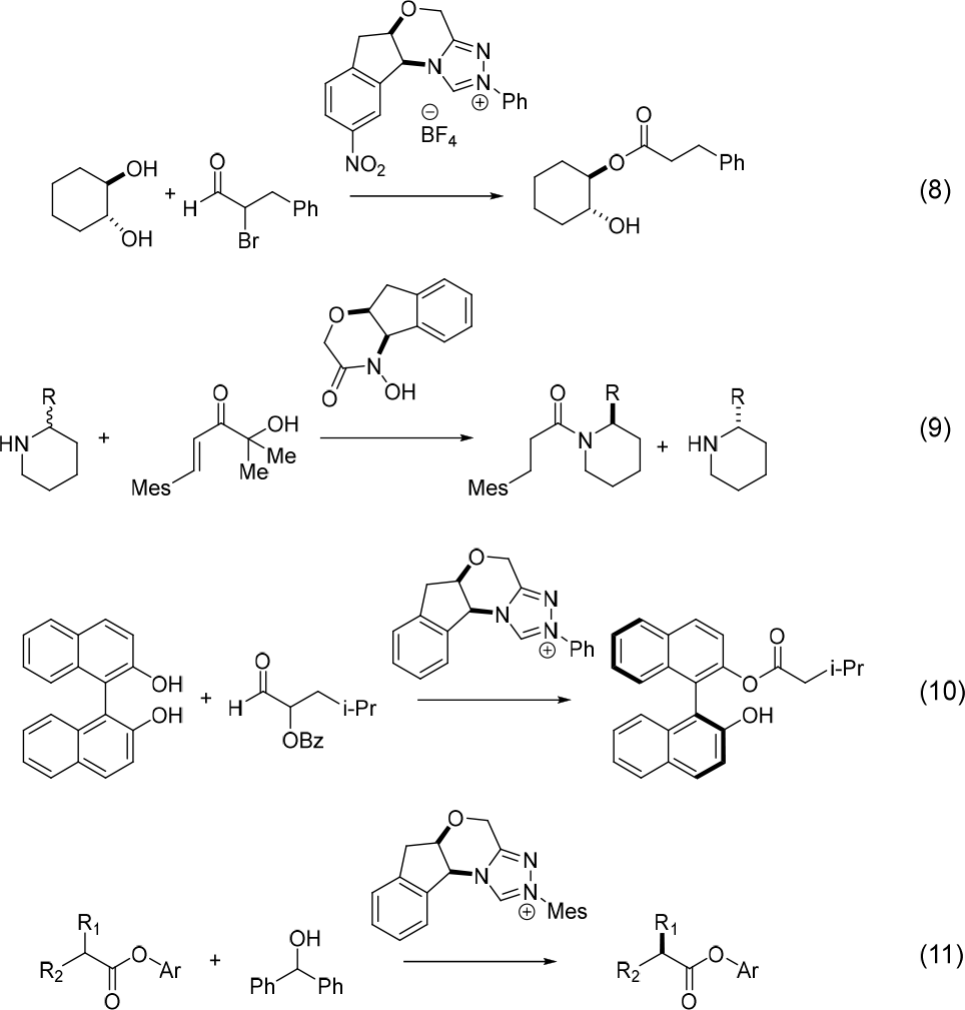
Three NHC-catalyzed KRs and one DKR for which H-bond networks play key roles in stereoselectivity.

**Figure 8:**
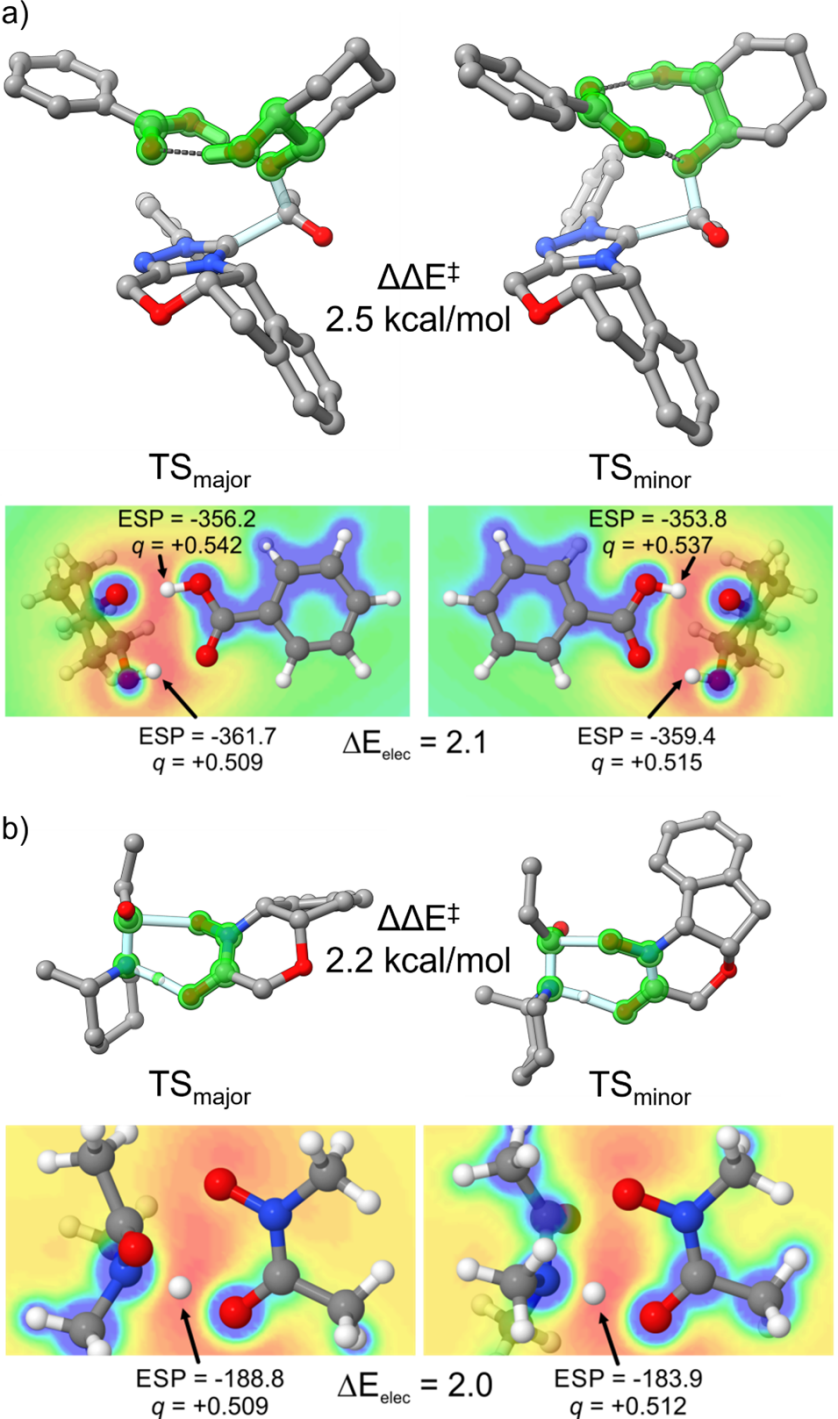
Stereocontrolling TS structures for a) reaction 8 from Yamada et al.^54^ and b) reaction 9 from Kozlowski et al.^55^ along with the quantification of the electrostatic stabilization of the key proton(s) within the H-bonding networks (highlighted) that underlie the stereoselectivity. Selected hydrogens removed for clarity. Portions adapted with permission from ref (3). Copyright (2017) American Chemical Society.

Our foray into this area was directed toward understanding the role of protic additives and the origin of the selectivity in a series of NHC-catalyzed KRs. Across three disparate examples of KRs (two of which had previously been studied computationally),^54,55^ together with one case of dynamic kinetic resolution (DKR), we identified electrostatic interactions as the universal driver of selectivity (reactions 8–11, Figure 7).^3^ First, we showed that steric interactions play a small role in the observed selectivities. Instead, we found that the stereocontrolling TS structures in these four reactions feature hydrogen bonding networks with markedly different features yet always provide greater stabilization of the favored structure. For example, reaction 8 has a cyclic OH···O interaction involving a benzoic acid additive that is critical but not directly involved in the critical bond-forming processes (see Figure 8a), whereas for reaction 9, the vital hydrogen bond network is directly involved in the bond-forming/breaking step (see Figure 8b). Despite the different natures of these H-bond networks, fragmentation analysis indicates that the difference in energy of these H-bond networks between the major and minor TS structures is the main determinant of the difference in free energy barriers. Moreover, the protons involved in these H-bond networks are consistently in more favorable electrostatic environments in the TS structures, leading to the major stereoisomer, providing a simple electrostatic model that explains the observed selectivity in all four transformations. This can be seen in Figure 8, where we quantify the electrostatic stabilization of the key proton(s) in reactions 8 and 9. In the former case, the two protons involved in the H-bond network bear similar partial charges; however, both protons experience more stabilizing ESPs in the major TS than the minor TS, resulting in a 2.1 kcal/mol contribution to the free energy barrier. Similarly, the transferring proton in reaction 9 experiences an ESP nearly 5 kcal/mol more negative in TS_major_ than TS_minor_, contributing 2.0 kcal/mol to the difference in TS free energies.

### 5.3 Counteranion Catalysis

**Figure 9:**
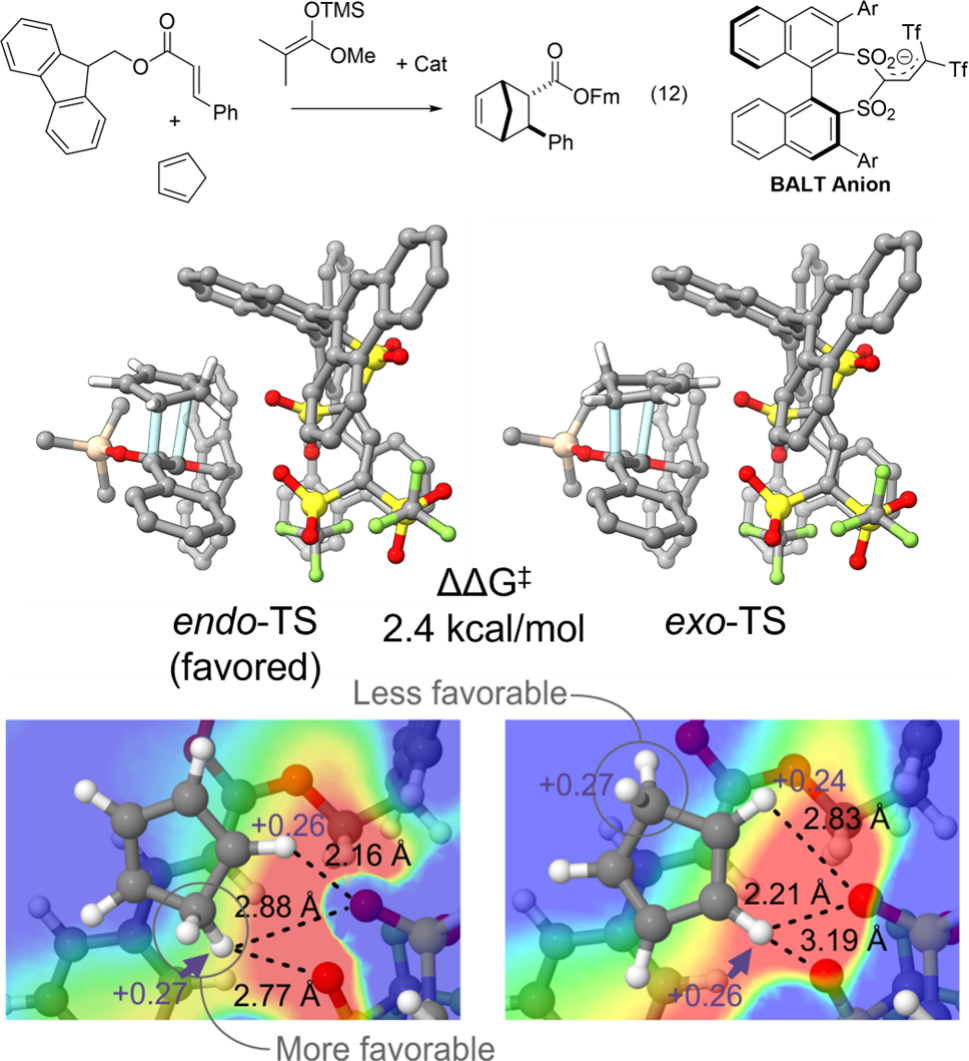
Asymmetric silylium ion-catalyzed Diels–Alder cycloaddition of cinnamate esters to cyclopentadiene from List et al.,^57^ along with representative *endo*- and *exo*-TS structures. The ESP due to the catalyst and dienophile in the plane of key CP hydrogen atoms is shown for the endo (left) and exo (right) TS structures, for one example, along with natural atomic charges for selected H atoms and interaction distances. Selected hydrogens removed for clarity. Portions adopted with permission from ref (4). Copyright (2016) John Wiley and Sons.

Chiral counteranion catalysis, or asymmetric counterion-directed catalysis (ACDC), is a focal point for asymmetric method development,^56^ yet our mechanistic understanding lags significantly behind ongoing methodological advances. This is due at least in part to the large size of the catalysts, which places these systems at the limits of what can be readily handled with DFT. This complexity is further exacerbated when the counteranion lacks obvious substrate recognition sites since there will be an enormous number of potential TS geometries varying in terms of the conformations of the substrate and catalyst as well as the precise arrangement of these species in the complex. We addressed one such challenge by studying^4^ four examples of asymmetric silylium ion-catalyzed Diels–Alder cycloadditions of cinnamate esters to cyclopentadiene (CP) from List and co-workers^57^ (reaction 12, Figure 8). This reaction can yield four potential stereoisomers, arising from either the *endo* or *exo* addition of CP to the two faces of the cinnamate ester. The *endo*:*exo* ratio of this reaction exceeds 13:1 for all four systems examined regardless of the enantioselectivity, implying a consistently large gap in free energy between the corresponding *endo* and *exo* transition states. While we found that dispersion interactions play a role in many aspects of this reaction, including the geometry of the preferred binding mode, the energy difference between the *endo* and *exo* transition states is primarily electrostatic in origin. In particular, the structures of the lowest-lying *exo*- and *endo*-TSs are nearly identical, apart from the CP orientation. However, the partial charges of the CP hydrogen atoms are not uniform, with the CH_2_ hydrogens carrying relatively more positive charge compared with their CH counterparts (Figure 9). Thus, the different CP poses lead to distinctive electrostatic environments for the methylene group, which enjoys greater electrostatic stabilization in the *endo*-TS than in the *exo*-TS.

## 6 Outlook

Through representative examples, we have illustrated electrostatic interactions as key modulators of reactivity and selectivity across a broad spectrum of organocatalytic reactions. Computational analysis of organocatalytic reactions is most often relegated to the role of rationalizing experimental results retrospectively; true predictions are rare. Reactions that hinge on electrostatic effects are one area where computational chemistry could prove highly effective in terms of prospective catalyst design due to the utility of computations to provide atomic-level details of electrostatic interactions in TS structures. Of course, the impact of electrostatic interactions on reactivity and selectivity transcends the area of organocatalysis, with crucial involvement also documented in metal catalysis, Lewis acid catalysis, photoredox catalysis, supramolecular catalysis, and biocatalysis. In organometallic catalysis, Schoenebeck and co-workers^58^ leveraged the electronegativity of CF_3_ substituents when designing a ligand to trigger the demanding reductive elimination of ArCF_3_ from Pd(II), by inducing electrostatic repulsion of the “leaving” CF_3_ group. In a major success story of computationally guided design, Head-Gordon and co-workers^59^ recently improved the efficiency of a *de novo* designed Kemp eliminase enzyme by modulating the local electrostatic environment in the active site. Such examples attest to the potential of a computationally directed design based on electrostatics.

Despite this considerable promise, the full power of electrostatically driven reactivity and selectivity has yet to be fully harnessed. We suggest untapped potential in three particular areas. First, electric fields within enzyme binding pockets are often computed and analyzed, and the importance of electric fields in steering biocatalytic reactions and transition metal catalyzed reactions is well recognized.^60,61^ Similar analyses have rarely been applied to the realm of organocatalysts. However, many of the above discussions based on ESPs can be recast in the language of electric fields (the electric field is the negative of the gradient of the ESP), potentially providing greater insight and avenues for catalyst design. For instance, while we discussed Matile’s anion-π-catalyzed Kemp elimination in terms of the stabilization of negative charge by the ESP due to the NDI (Figure 2), we could instead consider the movement of the proton relative to the electric field created by the NDI. In the case of the original catalyst (**1**), the proton is moving in a region of minimal electric field strength, whereas in the redesigned catalyst (**2**), there is a strong electric field that drives the proton from the substrate to the carboxylate, driving the reaction. Similarly, our finding that the precise placement of key protons within the heterogeneous electrostatic environment of CPA catalysts could be analyzed in terms of the electric fields experienced by these protons.

Second, current experimental endeavors in catalysis rely on the stabilization of the TS by a preinstalled and permanently charged motif in the substrate or catalyst. As the field advances, it may be possible to design catalysts with transient electrostatic directing groups or even to tune the electrostatic environments distant from the catalytic center. Despite the conceptual complexity involved, recent efforts by Phipps et al.^62^ have provided a significant step forward in this direction.

Finally, integration of more traditional DFT-based studies with data science and machine learning may constitute another avenue for innovation,^63−65^ as exemplified by the use of secondary-sphere electrostatic interactions to fine-tune organocatalyst reactivity by Milo and co-workers.^66^ Such strategies will be particularly useful for the rational design of organocatalysts, given the high computational demand associated with their large size and flexibility.^67^ Our efforts to design catalysts for asymmetric propargylations through automated quantum chemical predictions^68^ has shown that enhanced selectivity can be achieved by modulating the ESP of the organocatalyst through fluorination. Such emerging techniques offer a new frontier for reaction discovery.
